# Recent progress in the direct monofluoromethylation of C–H bonds

**DOI:** 10.1039/d5ra10069k

**Published:** 2026-07-07

**Authors:** Shahrzad Abdolmohammadi, Somayeh Soleimani-Amiri, Widad Ibrahem Yahya, Ayten A. Niyazova, Ali Z. Zalov, Huseyn Imanov, Huseynova Afet Temir, Esmail Vessally

**Affiliations:** a Department of Chemistry, ST.C., Islamic Azad University Tehran Iran sh.abdolmohammadi@iau.ac.ir; b Department of Chemistry, Ka.C., Islamic Azad University Karaj Iran so.soleimani@iau.ac.ir; c Chemistry Department, College of Sciences, Kufa University Najaf Iraq; d Composite Materials Scientific Research Center, Azerbaijan State University of Economics (UNEC) 194 M.Mukhtarov Str. Baku Azerbaijan; e Department of Analytical and Organic Chemistry, Azerbaijan State Pedagogical University Azerbaijan; f Department of Chemistry, Faculty of Natural Sciences and Agriculture, Nakhchivan State University Nakhchivan Azerbaijan; g Department of Organic Chemistry, Baku State University Baku Azerbaijan; h Department of Chemistry, Payame Noor University Tehran Iran

## Abstract

Fluorine chemistry is of critical importance in the agricultural, pharmaceutical, and materials industries, as the incorporation of fluorine atoms or fluorine-containing groups into organic compounds can significantly enhance their chemical and physical properties. Among these groups, the monofluoromethyl (CH_2_F) group has emerged as a distinctive fluorine motif, serving as a metabolically stable and lipophilic bioisostere for functional groups such as NH_2_, OH, and SH, and making it a privileged structural unit in drug discovery. As a result, considerable attention has recently been directed toward developing new and practical methods for introducing this group into organic molecules. In this regard, the direct monofluoromethylation of C–H bonds has recently emerged as an ideal strategy, as it does not require pre-functionalized starting materials and allows for more efficient and streamlined synthetic routes. This review highlights recent advances in this area, with particular emphasis on the mechanistic aspects of the reactions.

## Abstract 2

This review highlights recent advances in the direct monofluoromethylation of C–H bonds, with emphasis on catalytic methods, reaction mechanisms, substrate scope, and synthetic applications.

## Graphical abstract

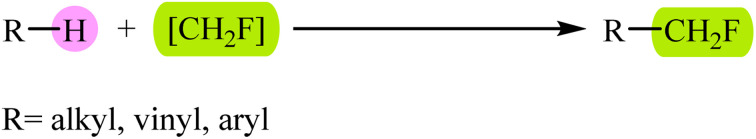

## 1. Introduction

**Scheme 1:**
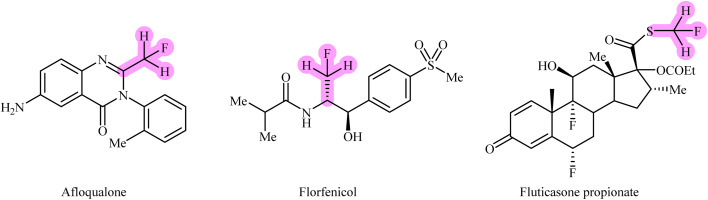
Examples of approved drugs featuring a CH_2_F group.

The incorporation of fluorine motifs into organic architectures has become increasingly important for optimizing the pharmacological profiles of bioactive compounds.^1–3^ Among the various fluorine-containing motifs, fluoromethyl groups such as trifluoromethyl (CF_3_), difluoromethyl (CF_2_H), and monofluoromethyl (CH_2_F) have attracted significant interest, reflected by their frequent occurrence in numerous approved drugs.^4–6^ Notably, the CH_2_F group functions as a metabolically stable and lipophilic bioisostere of functional groups such as NH_2_, OH, and SH. This motif is represented in a number of natural products and synthetic pharmaceuticals (Scheme 1),^7,8^ and is also incorporated into various positron emission tomography (PET) radiotracers, including inpyrfluxam, mefway, and fluorocholine.^9^ Beyond medicinal chemistry, this motif has begun to find applications in materials science; for instance, Liu and co-workers recently demonstrated that its incorporation into silicone rubber considerably improves stain resistance and slip properties.^10^ Despite substantial progress in trifluoromethylation and difluoromethylation over the past decades,^11,12^ the development of efficient and general methods for monofluoromethylation remains a significant and largely unmet challenge.^13^ Traditionally, monofluoromethylation has relied on electrophilic introduction of the CH_2_F unit using fluoromethylating agents of the general formula CH_2_FX (X = Cl, Br, I, OH). Subsequently, a variety of CH_2_F transfer reagents operating *via* nucleophilic or radical pathways have also been developed, expanding the repertoire of available monofluoromethylation strategies.^7^ However, most reported examples across these three regimes still depend on pre-functionalized starting materials. Accordingly, direct monofluoromethylation *via* C–H activation represents an ideal yet particularly challenging approach.

**Fig. 1:**
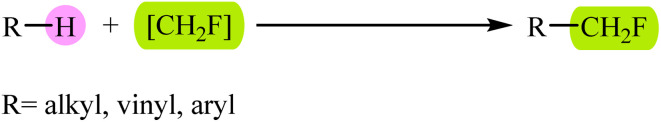
Direct monofluoromethylation of C–H bonds.

Recently, direct C–H functionalization has emerged as an ideal strategy for accessing structurally complex molecules from readily available substrates by directly cleaving and functionalizing C–H bonds.^14–18^ This approach avoids the need for pre-functionalized starting materials and thereby enhances the atom economy and step economy of organic synthesis.^19^ In this context, various strategies have been recently explored for the direct monofluoromethylation of C–H bonds, offering new opportunities for the efficient synthesis of CH_2_F-containing compounds (Fig. 1). Although several recent review articles have described advances in the direct introduction of fluorine-containing functional groups into organic molecules *via* C–H bond functionalization,^20^ as well as developments in monofluoromethylation reactions,^7,9,21^ none has specifically focused on the direct monofluoromethylation of C–H bonds. In this review we will highlight recent developments and achievements in this area, with particular emphasis on the mechanistic aspects of the reactions.

## 2. Direct monofluoromethylation of aliphatic C–H bonds

**Scheme 2:**
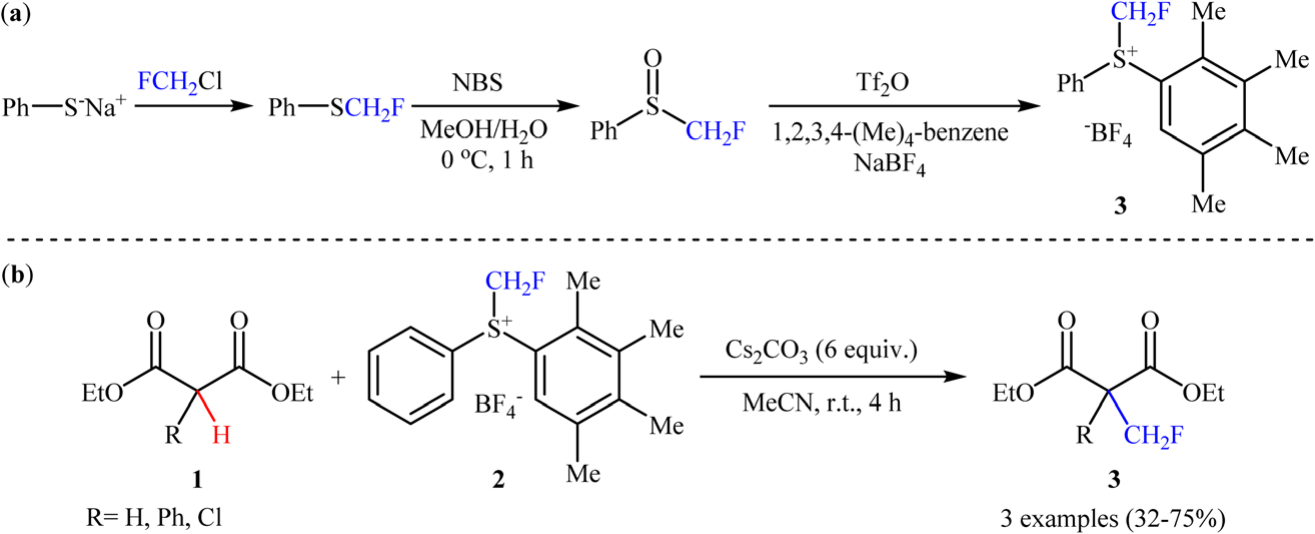
(a) Synthetic route for the preparation of *S*-monofluoromethyl-*S*-phenyl-2,3,4,5-tetramethylphenylsulfonium tetrafluoroborate 3; (b) Olah᾿s synthesis of CH_2_F-functionalized malonic esters 3.

In 2008, the group of Olah reported the preparation of a superelectrophilic monofluoromethylating reagent, *S*-monofluoromethyl-*S*-phenyl-2,3,4,5-tetramethylphenylsulfonium tetrafluoroborate 3, *via* a three-step procedure starting from inexpensive sodium thiophenolate.^22^ The synthesis involves nucleophilic substitution of liquefied CH_2_FCl with sodium thiophenolate, followed by oxidation of the resulting monofluoromethyl sulfide to the corresponding sulfoxide using *N*-bromosuccinimide (NBS), and a subsequent Friedel–Crafts-type reaction with 1,2,3,4-tetramethylbenzene in the presence of trifluoromethanesulfonic anhydride (Scheme 2a). By employing this electrophilic monofluoromethylating reagent, they investigated for the first time the possibility of direct monofluoromethylation of aliphatic C–H bonds. A small set of malonic esters 1 underwent monofluoromethylation upon treatment with *S*-(monofluoromethyl)diarylsulfonium tetrafluoroborate 2 under basic conditions, furnishing monofluoromethylated products 3 in moderate to good yields (Scheme 2b). The reaction also exhibited tolerance toward β-disulfones. Although the substrate scope was confined to carbon nucleophiles with sufficiently acidic C–H bonds, this work represents the first example of direct C(sp^3^)–H monofluoromethylation and served as an important foundation for subsequent advances in the field. Subsequently, the group of Shibata designed and synthesized a monofluoromethylsulfoxinium salt, [(oxido)phenyl(monofluoromethyl)-λ^4^-sulfanylidene]dimethylammonium tetrafluoroborate, which was applied as a monofluoromethylating reagent for the direct C(sp^3^)–H functionalization of β-ketoesters.^23^ Unexpectedly, exclusive *O*-monofluoromethylation was observed for all substrates examined. In contrast, use of the analogous trifluoromethylsulfoxinium salt with the same set of β-ketoesters resulted in selective *C*-trifluoromethylation, whereas the corresponding difluoromethylsulfoxinium salt afforded mixtures of *C*- and *O*-functionalized products. These results clearly demonstrate that the *C*/*O* regioselectivity in fluoromethylation of β-ketoesters using this class of sulfoxinium-based reagents is strongly dependent on the degree of fluorination of the fluoromethyl group, and is largely independent of the β-ketoester substrate structure, solvent, or base employed. Based on computational studies, the authors proposed that monofluoromethylation proceeds *via* nucleophilic attack of the enolate oxygen at the sulfur center of the reagent, forming a sulfurane-type intermediate that subsequently generates O˙ and ˙CFH_2_ radicals along with dimethylaminophenyl sulfinamide. In contrast, due to the electron-deficient nature of the CF_3_ group, the enolate preferentially attacks the more cationic trifluoromethyl carbon center of the reagent, leading to the *C*-alkylated product *via* an ionic S_N_2 pathway.

**Scheme 3:**
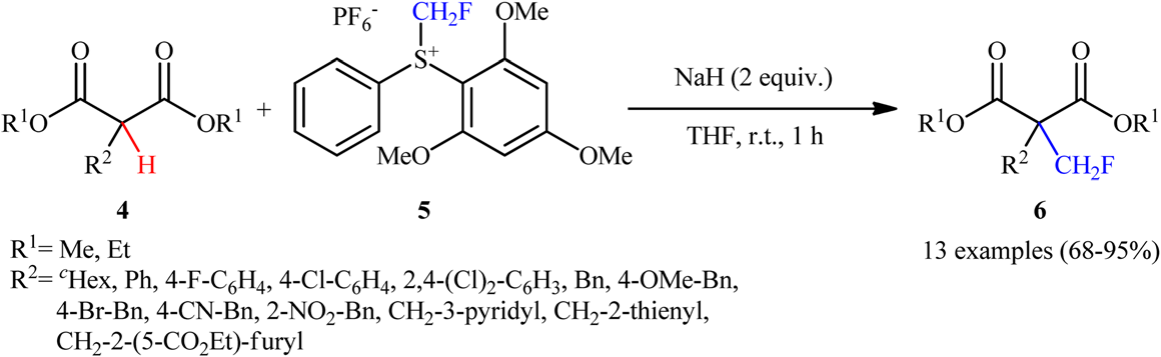
Liu᾿s direct C–H monofluoromethylation of malonic esters 6.

Following these works, in 2020, the Liu research group designed and synthesized a structurally related, bench-stable *S*-(monofluoromethyl)sulfonium salt 5 from phenyl monofluoromethyl sulfoxide and inexpensive 1,3,5-trimethoxybenzene.^24^ This reagent was subsequently applied as an efficient electrophilic monofluoromethylating agent for the direct C(sp^3^)–H functionalization of various malonic ester derivatives 4. Conducted at room temperature using NaH as the base, these reactions proceeded selectively to furnish the corresponding *C*-monofluoromethylated products 6 in good to excellent yields within 1 hour (Scheme 3). Notably, the methodology proved amenable to scale-up with only a slight loss in yield. In contrast to malonates, β-ketoesters produced a mixture of *C*- and *O*-monofluoromethylated regioisomers, with the *O*-product predominating. Screening different bases revealed that Cs_2_CO_3_ favored *O*-selectivity, affording products with an excellent *O*/*C* ratio. These results clearly indicate that, as in alkylation reactions in general, the *C*/*O* regioselectivity of monofluoromethylation depends not only on the nature of the enolate substrate but also on the reactivity of the alkylating reagent and the reaction conditions, such as the base and solvent.

**Scheme 4:**
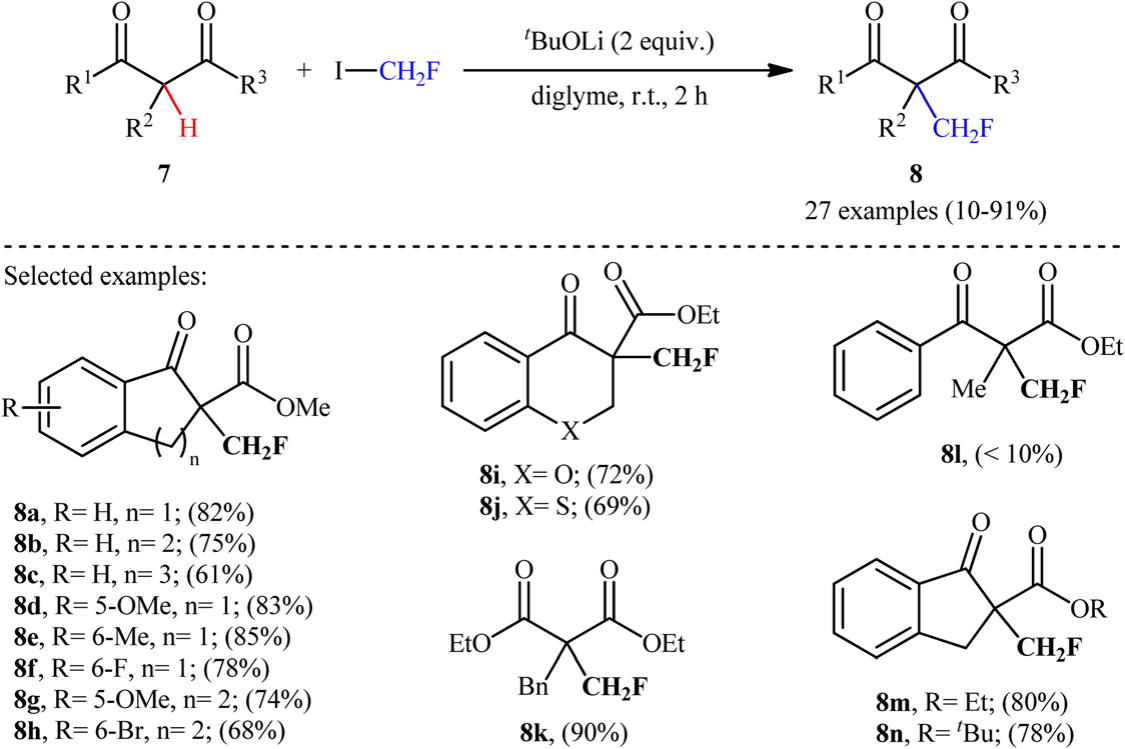
α-Monofluoromethylation of β-ketoesters 7 with ICH_2_F.

**Fig. 2:**
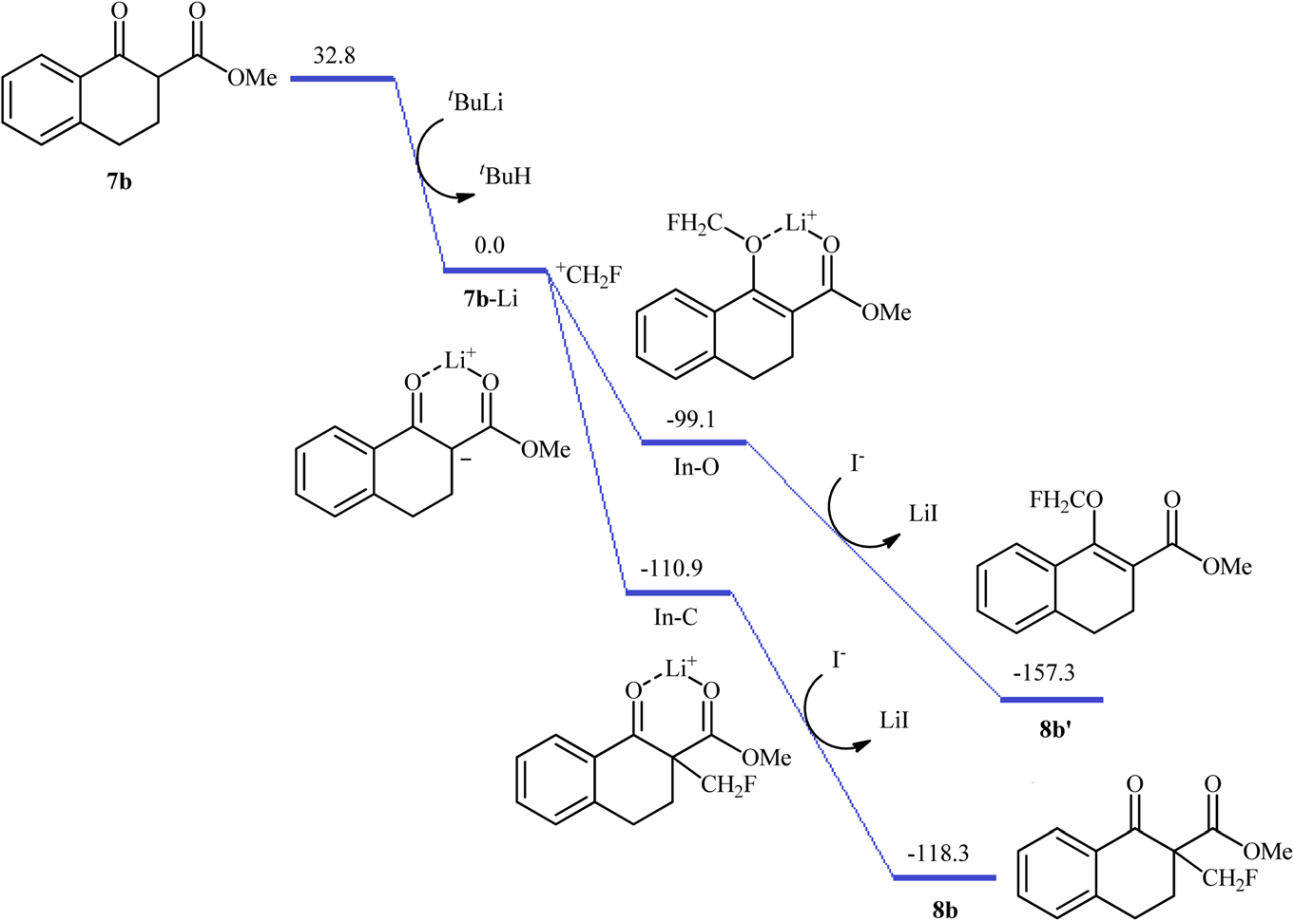
Computed energy profile (Δ*G*, kcal mol^−1^) at the M06-2X/6-311G* level of theory for the formation of *C*- and *O*-monofluoromethylated products.

In this context, Jiang and co-workers developed an efficient strategy for the selective α-monofluoromethylation of challenging β-ketoesters, employing commercially available fluoroiodomethane (ICH_2_F) as an easy-to-handle monofluoromethylating agent, and ^*t*^BuOLi as the base.^25^ The procedure was shown to be general and a wide range of cyclic β-ketoester derivatives 7 participated in the reaction (Scheme 4). Malonic esters were also successfully functionalized under the same conditions; however, acyclic β-ketoesters reacted sluggishly under this protocol. It should be mentioned that alongside the desired *C*-monofluoromethylated products 8, minor amounts of *O*-monofluoromethylated byproducts were consistently detected in all cases. To gain insight into the mechanism of this carbon-selective α-monofluoromethylation, the authors performed density functional theory (DFT) calculations (Fig. 2), which indicate that the reaction proceeds *via* a direct electrophilic substitution pathway, with the *C*-functionalized product being thermodynamically favored over the *O*-functionalized analogue. As shown in Fig. 2, the relative free energy of the intermediate In–C, corresponding to electrophilic attack at the carbon atom, is 11.8 kcal mol^−1^ lower than that of In–O, arising from electrophilic attack at oxygen. Moreover, the monofluoromethylated product 8b is more stable than its *O*-functionalized counterpart 8b′ by 24.0 kcal mol^−1^, consistent with the observed *C*/*O* regioselectivity ratio of 90 : 10.

**Scheme 5:**
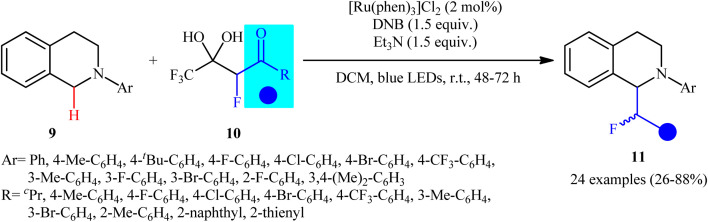
Zhu᾿s synthesis of 1-(fluoromethyl)-tetrahydroisoquinolines 11.

In a notable advance, Zhu and co-workers reported a visible-light photoredox-catalyzed direct C(sp^3^)–H monofluoromethylation of tetrahydroisoquinolines under ambient conditions, employing stable β-fluorinated *gem*-diols that were readily generated from the corresponding trifluoroacetylacetone derivatives *via* reaction with Selectfluor.^26^ An extensive optimization study was conducted using *N*-phenyl-substituted tetrahydroisoquinoline and 2,4,4,4-tetrafluoro-3,3-dihydroxy-1-phenylbutan-1-one as model substrates, during which the effects of photocatalysts, oxidants, bases, and solvents were carefully examined. The optimal conditions were identified as 2 mol% [Ru(phen)_3_]Cl_2_ in the presence of 1.5 equivalents of Et_3_N and *meta*-dinitrobenzene (DNB), with DCM emerging as the most suitable solvent among those examined (*e.g*., DCM, DCE, MeCN, THF, DMF, and DMSO). Under these conditions, irradiation with blue LEDs enabled a wide range of *N*-substituted tetrahydroisoquinolines 9 to undergo selective C1-monofluoromethylation with β-fluorinated *gem*-diols 10, delivering the corresponding 1-(fluoromethyl)tetrahydroisoquinolines 11 in good to excellent yields (Scheme 5). In terms of substrate scope, both electron-rich and electron-poor aryl-substituted tetrahydroisoquinolines were well tolerated, although electron-rich substrates generally afforded higher yields than electron-poor ones. The scalability of the method was also demonstrated by the preparation of 2-fluoro-1-phenyl-2-(2-phenyl-1,2,3,4-tetrahydroisoquinolin-1-yl)ethanone on a 1.4 g scale in 85% yield. However, alkyl-substituted tetrahydroisoquinolines and the effect of blocking at the C1 position were not explored.

## 3. Direct monofluoromethylation of olefinic C–H bonds

**Scheme 6:**
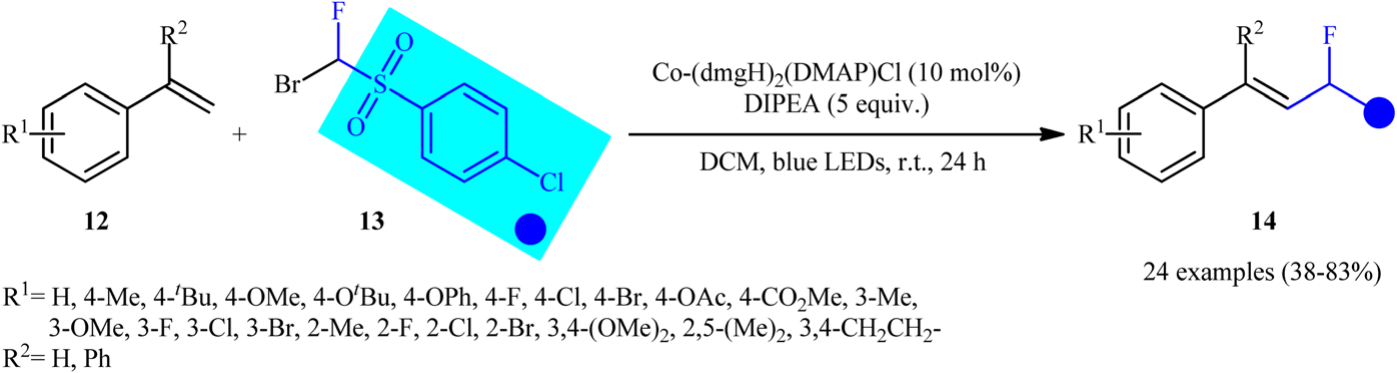
Wu᾿s synthesis of allylic monofluoromethylated compounds 14.

**Scheme 7:**
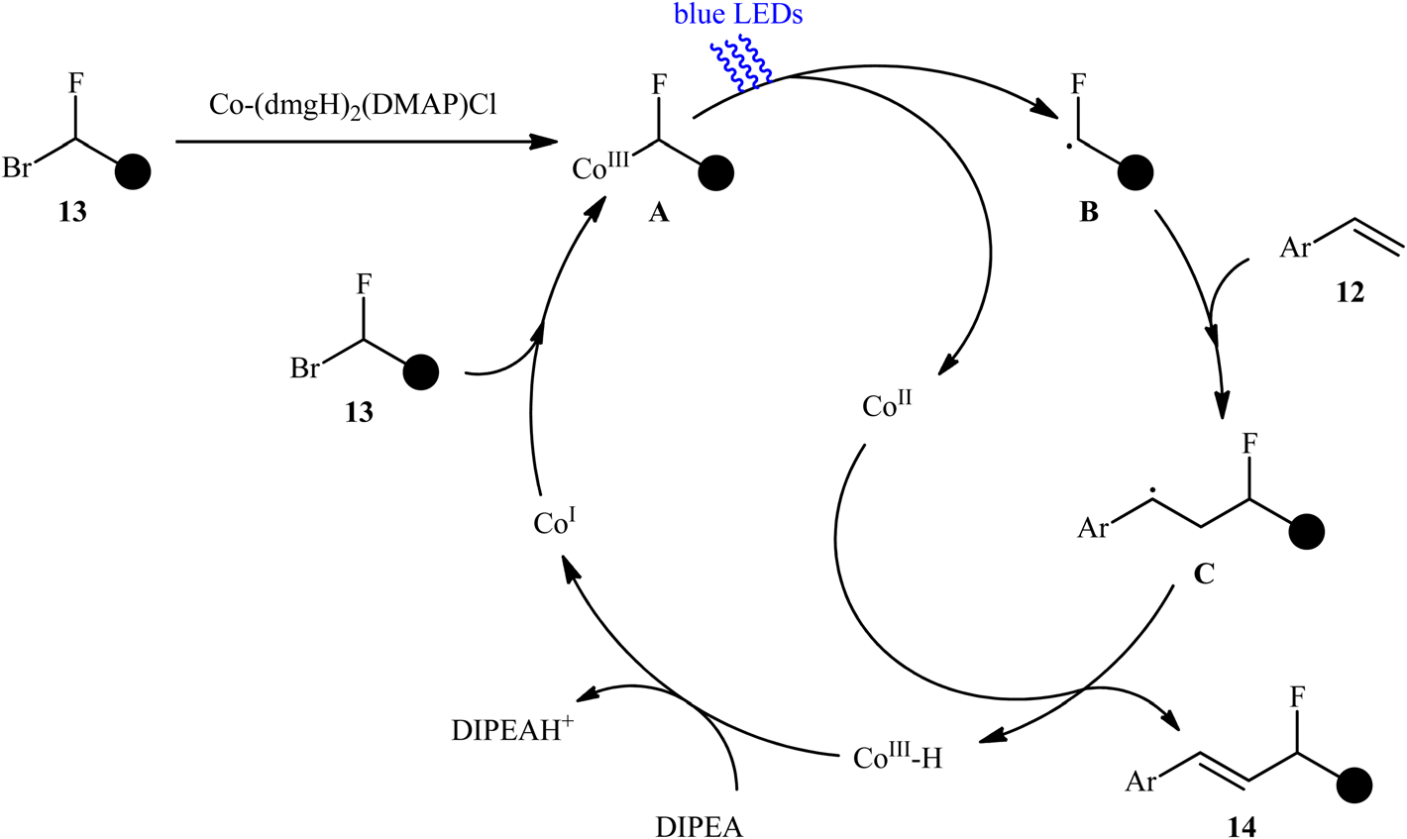
Proposed mechanism for the formation of allylic monofluoromethylated compounds 14.

To the best of our knowledge, no general methodology has been reported for the direct monofluoromethylation of olefinic C–H bonds, and only a single, very recent example describing the direct introduction of a functionalized monofluoromethyl group into C_(alkenyl)_–H bonds has been disclosed by Wu and co-workers.^27^ In this report they demonstrated that the reaction between styrene derivatives 12 with sulfonyl monofluoromethyl bromide 13 as the fluoromethylating agent under visible-light irradiation in the presence of a cobaloxime catalyst delivered the desired allylic monofluoromethylated products 14 in acceptable yields (Scheme 6). The transformation displayed a clear dependence on the electronic nature of the substrates, with electron-rich derivatives reacting more efficiently than electron-poor analogues (80% yield for the 4-methoxy-substituted substrate compared to 38% for the 4-methoxycarbonyl derivative). To further demonstrate the synthetic utility of this methodology, the authors successfully applied it to the allylic monofluoromethylation of a variety of bioactive molecule derivatives, including those derived from estrone, thymol, amino acids, glucose, and ciprofibrate. Notably, a broad range of functional groups, including fluoro, chloro, bromo, ether, ester, and ketone functionalities, were well tolerated under the reaction conditions, making this transformation a versatile synthetic handle for further derivatization. However, the applicability of neither internal styrenes nor aliphatic alkenes as starting materials was investigated under these conditions. The authors demonstrated that the sulfonyl group can be efficiently removed in a single-step procedure by treatment with magnesium in methanol to give the monofluoromethylated products in high yields. With respect to the proposed mechanism (Scheme 7), the authors suggested that the cobalt catalyst first reacts with reagent 13 to generate intermediate A. Upon irradiation with visible light, homolytic cleavage of the C–Co bond occurs, producing a Co^II^ species and a monofluoromethyl radical B. The latter adds to alkene substrate 12 to form alkyl radical intermediate C, which subsequently undergoes *β*-hydride elimination to deliver the allylic monofluoromethylated product 14. In parallel, the Co^II^ species abstracts a hydrogen atom to afford a Co^III^–H intermediate, which is reduced under the basic conditions to give a Co^I^ species. The catalytic cycle is closed by an S_N_2-type reaction between the Co^I^ species and monofluoromethylating reagent 13, regenerating the active cobalt intermediate A.

## 4. Direct monofluoromethylation of (hetero)aromatic C–H bonds

### 4.1. Catalyst-free reactions

**Scheme 8:**
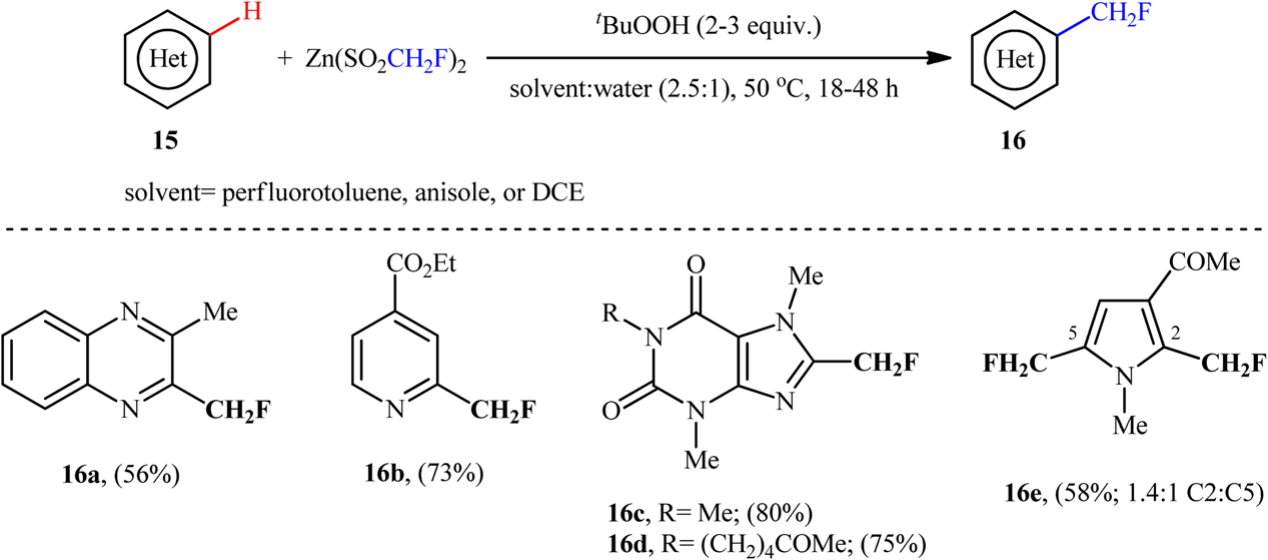
Baran᾿s synthesis of monofluoromethylated heteroarenes 16.

The feasibility of direct monofluoromethylation of (hetero)aromatic C–H bonds was first reported by Baran and co-workers,^28^ who showed that treatment of nitrogen-rich heterocyclic compounds 15 with bench-top-stable zinc monofluoromethanesulphinate Zn(SO_2_CH_2_F)_2_ in the presence of excess ^*t*^BuOOH in a suitable aqueous–organic solvent resulted in the formation of corresponding monofluoromethylated heteroarenes 16 (Scheme 8). The transformation proceeds *via* monofluoromethyl radicals, which preferentially add to electron-poor sites of (hetero)arenes, likely because of the electrophilic character of monofluoromethyl radicals. Although the reactions provided good isolated yields, the substrate scope was limited and regioselectivity remained moderate. Notably, a similar principle was also successfully applied to the direct di- and tri-fluoromethylation of same set of *N*-heteroarenes.

**Scheme 9:**
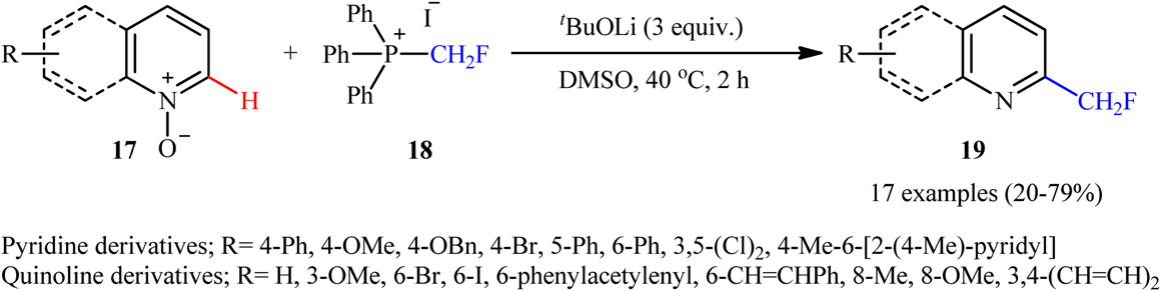
Qing᾿s synthesis of C2-fluoromethylated pyridine and quinoline derivatives 19.

**Scheme 10:**
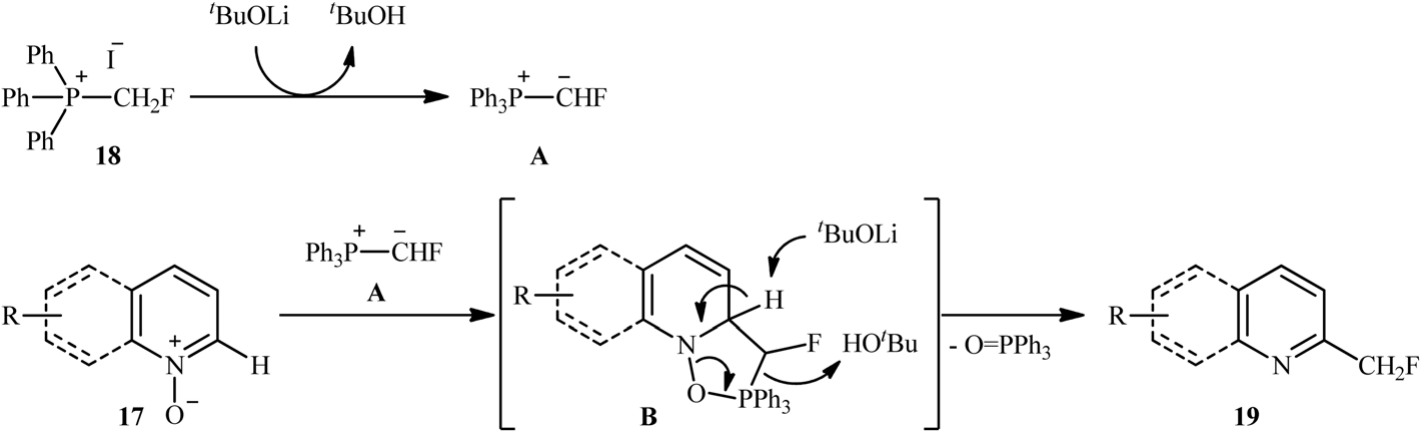
Suggested mechanism for the formation of C2-fluoromethylated *N*-heteroarenes 3.

Following this work, Qing and co-workers prepared (fluoromethyl)triphenylphosphonium iodide 18 from stoichiometric Ph_3_P and CH_2_FBr, and demonstrated its use as a CH_2_F source in the site-selective monofluoromethylation of six-membered heteroaryl *N*-oxides 17 under mild, catalyst-free conditions.^29^ Among the various common organic solvents like THF, DMSO, MeCN, toluene; DMSO was the most efficient for this reaction. Under the optimized reaction conditions (^*t*^BuOLi, DMSO, 40 °C), the corresponding C2-fluoromethylated pyridine and quinoline derivatives 19 were obtained in poor to high yields (Scheme 9). Substrates bearing synthetically useful functional groups, including vinyl, alkynyl, bromo, and iodo substituents at various positions, were well tolerated. Notably, a structurally complex substrate derived from roflumilast, a drug used to treat chronic obstructive pulmonary disease, was also successfully subjected to the standard reaction conditions, affording the corresponding fluoromethylated roflumilast analogue in moderate yield. However, 2,6-di-substituted pyridine *N*-oxides and 2-substituted quinoline *N*-oxides were unreactive under the standard conditions. A plausible reaction mechanism for the formation of C2-fluoromethylated *N*-heteroarenes 19 is outlined in Scheme 10. Deprotonation of (fluoromethyl)triphenylphosphonium iodide 18 by ^*t*^BuOLi initially generates fluoromethylene phosphonium ylide A. Subsequent intermolecular [3 + 2] annulation between ylide A and heteroaryl *N*-oxides 17 furnishes intermediate B, which undergoes base-promoted aromatization to afford the C2-fluoromethylated products 19.

### 4.2. Metal-catalyzed reactions

**Scheme 11:**
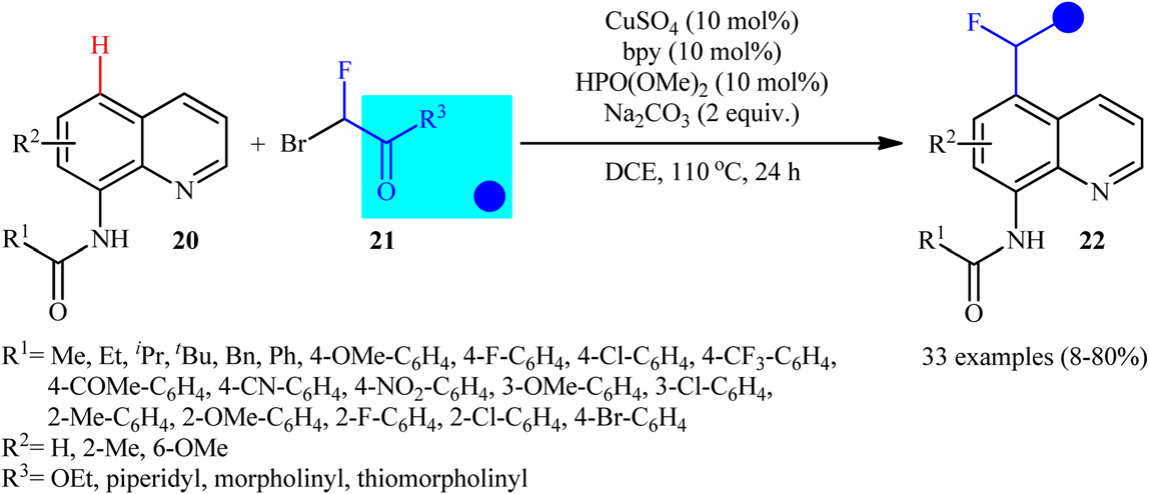
Wu᾿s synthesis of 5-(fluoromethyl)quinolin-8-amines 22.

**Scheme 12:**
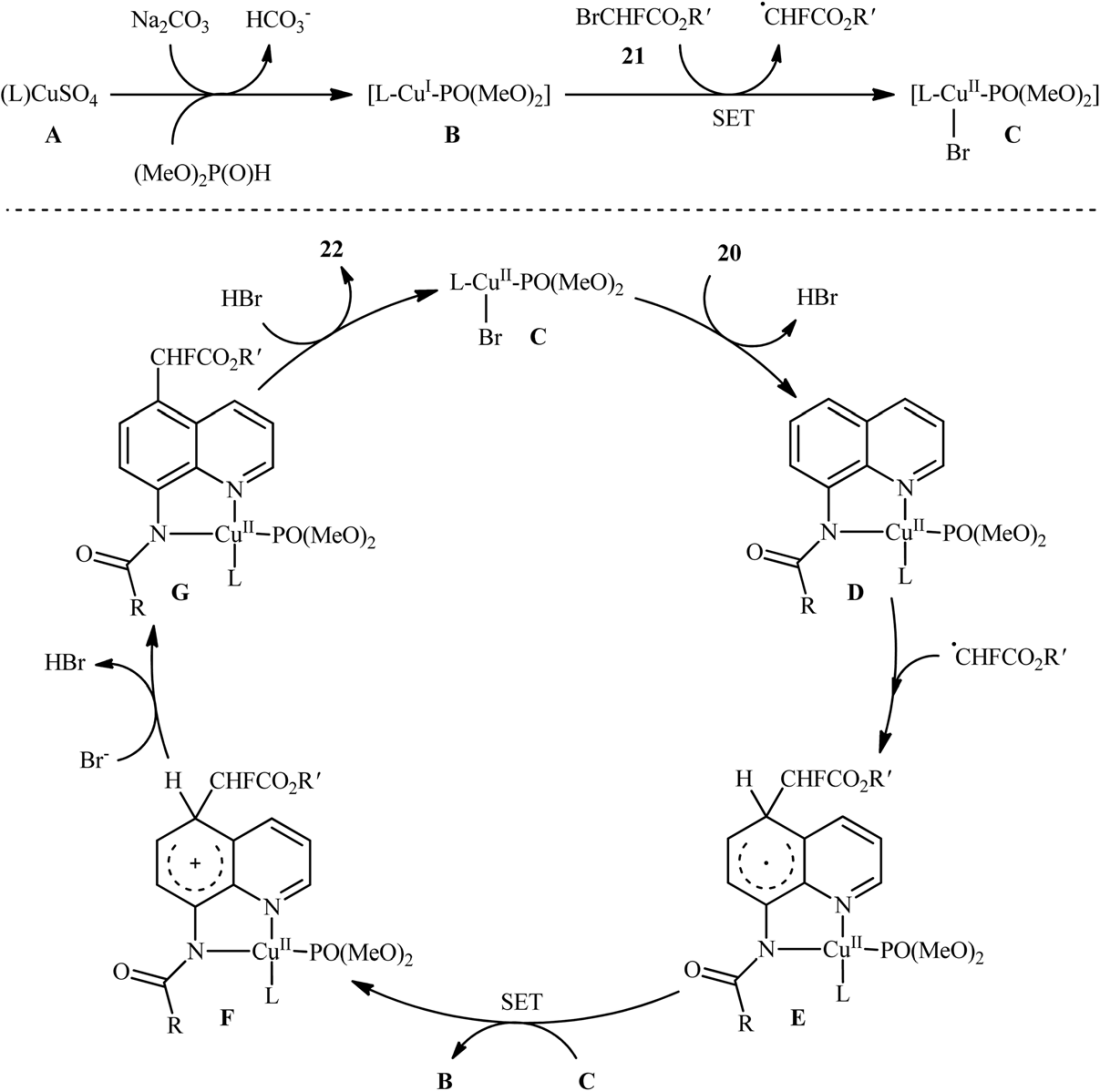
Possible mechanism for the formation of 5-(fluoromethyl)quinolin-8-amines 22.

In 2019, Wu and co-workers disclosed one of the earliest metal-catalyzed approaches for the direct monofluoromethylation of (hetero)aromatic C–H bonds. Under the reported conditions, 8-aminoquinoline derivatives 20 underwent highly site-selective C5 monofluoromethylation upon reaction with α-bromo-α-fluoro carbonyl compounds 21, employing a CuSO_4_/2,2′-bipyridine (bpy)/HPO(OMe)_2_/Na_2_CO_3_ catalytic system in DCE at 110 °C.^30^ Using this protocol, 33 examples of 5-(fluoromethyl)quinolin-8-amines 22 bearing a variety of synthetically important functional groups at different positions were synthesized in yields ranging from poor to high (Scheme 11). The results clearly demonstrated that electron-rich substrates afforded significantly higher yields than their electron-poor counterparts. In addition to CuSO_4_, other copper sources, including CuCl, CuBr, CuI, CuCl_2_, CuBr_2_, CuSO_4_, Cu(OTf)_2_, and Cu(acac)_2_, were also capable of promoting the reaction, albeit with reduced efficiency. Notably, the presence of all catalyst components was crucial for the success of the reaction, as omission of any one of them resulted in extremely low yields or complete suppression of product formation. Based on a series of control experiments and precedents from the literature, the authors proposed that this transformation proceeds through a radical pathway, as depicted in Scheme 12. Initially, copper salt A is reduced by (OMe)_2_P(O)H in the presence of a base to generate the Cu(i)-[PO(OMe)_2_] species B. This species undergoes a single-electron transfer (SET) process with BrCHFCO_2_R 21 to produce the electrophilic fluoroalkyl radical ˙CHFCO_2_R along with intermediate C. Afterwards, coordination of C with 8-aminoquinoline 20 affords the chelated intermediate D. Subsequent capture of the ˙CHFCO_2_R radical by D affords the Cu(ii) intermediate E, which is converted into complex F through a SET process. A proton transfer step then furnishes intermediate G, which ultimately yields the product 22 while regenerating species C, thus completing the catalytic cycle. Shortly thereafter, a modified version of this protocol was employed by Gandelman and co-workers for the C2-selective direct monofluoroalkylation of benzoxazoles using 1-fluoro-1-haloalkanes as monofluoroalkylating agent.^31^

**Scheme 13:**
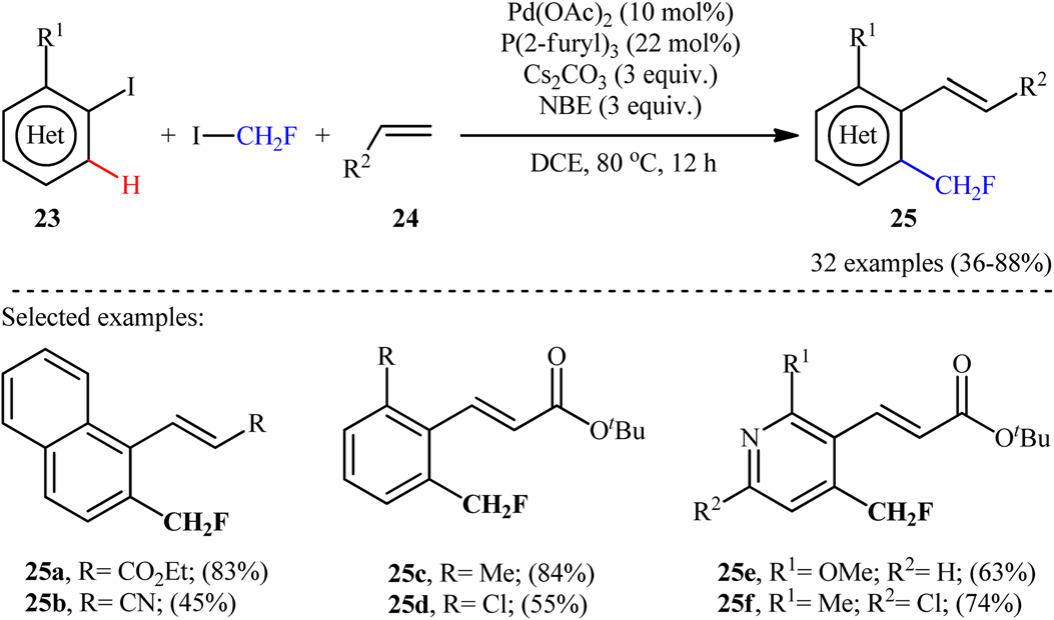
Li᾿s synthesis of polyfunctional fluoromethylated arenes 25.

Recently, Li and co-workers reported a robust three-component protocol for the synthesis of polyfunctional fluoromethylated arenes 25*via* a Pd-catalyzed sequential cross-coupling reaction of *ortho*-substituted iodoarenes 23, fluoroiodomethane (CH_2_IF), and terminal alkenes 24.^32^ The transformation was performed using a Pd(OAc)_2_/P(2-furyl)_3_/Cs_2_CO_3_/norbornene (NBE) catalytic system in DCE at 80 °C and exhibited a broad substrate scope, tolerating both iodoarenes and iodoheteroarenes bearing electron-donating or electron-withdrawing substituents, as well as a wide range of terminal alkenes, including α,β-unsaturated carbonyl compounds, styrenes, and acrylonitrile. The desired products were obtained in modest to high yields with excellent site-selectivity (Scheme 13). Mechanistically, it was proposed that the aryl iodide first undergoes oxidative addition to Pd(0), followed by norbornene insertion and *ortho* C–H activation to form a norbornyl palladacycle, which then reacts with CH_2_IF and an alkene coupling partner to furnish the final fluoromethylated product. Notably, the authors further expanded the substrate scope to aryl iodides lacking *ortho* substituents. Under these conditions, bis(fluoromethylated) internal styrenes were obtained exclusively, wherein selective monofluoromethylation occurred at the *ortho* C–H positions relative to the C–I bond and the alkenylation at the C–I bond.

### 4.3. Photo-catalyzed reactions

**Scheme 14:**
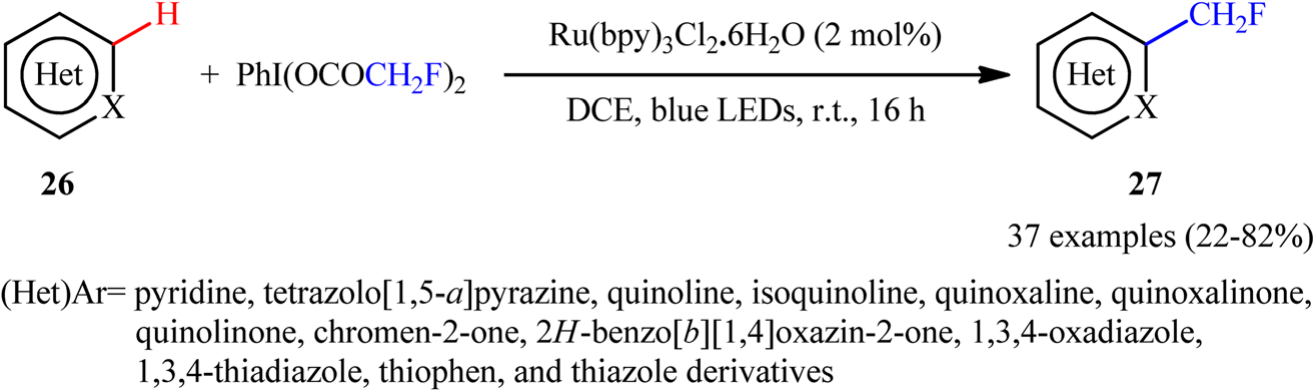
Photoredox-catalyzed direct C–H monofluoromethylation of heteroarenes 26 with PhI(OCOCH_2_F)_2_.

**Scheme 15:**
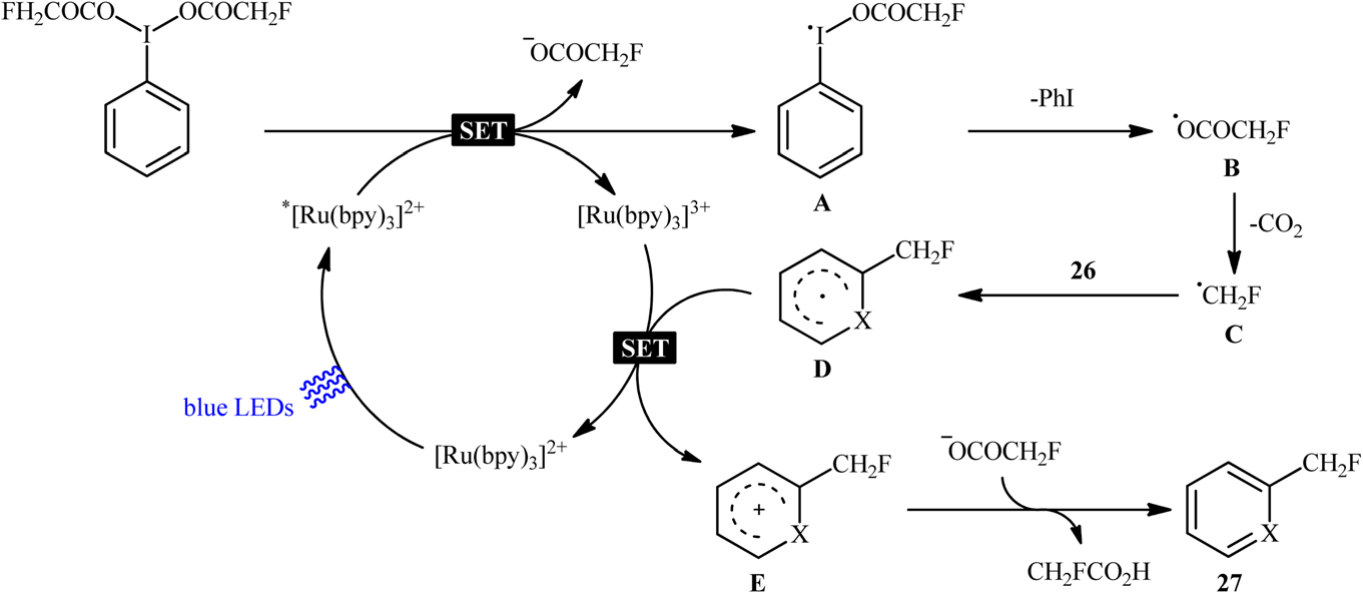
Mechanism that accounts the reaction in Scheme 14.

Inspired by the direct C–H monofluoroalkylation strategies developed by Qing and Zhao using ethyl bromofluoroacetate and diethyl 2-bromo-2-fluoromalonate under visible-light photoredox conditions,^33,34^ Oseka and Veliks along with their co-workers reported the first general protocol for photoredox-catalyzed direct C–H monofluoromethylation of heteroarenes using the non-commercial but readily accessible phenyliodine(iii) di(2-fluoroacetate) [PhI(OCOCH_2_F)_2_] as fluoromethylating reagent.^35^ In this study, various photocatalysts and solvents were examined and the Ru(bpy)_3_Cl_2_·6H_2_O/DCE system was found to be optimal for this reaction. Under these optimized conditions, a broad range of heteroaromatic substrates 26 underwent efficient fluoromethylation to furnish the corresponding products 27 in fair to high yields (Scheme 14). Furthermore, the authors demonstrated the use of their methodology for the late-stage functionalization of heterocycles containing biological and pharmaceutical active molecules such as piperonylic acid, vitamin E, estrone, (−)-menthol, caffeine, d-α-tocopherol, lawsone methyl ether, osthol, hymecromone, fasudi, and roflumilast. Based on control experiments and prior literature, the authors proposed a plausible mechanistic pathway for this reaction (Scheme 15). Upon visible-light irradiation, the photocatalyst is excited to generate the [Ru^II^]* species. Subsequent single-electron transfer (SET) between [Ru^II^]* and PhI(OCOCH_2_F)_2_ generates radical intermediate A along with Ru^III^. Next, homolytic cleavage of the I–O bond in radical A produces the fluoroacetoxy radical B, which undergoes decarboxylation to generate the monofluoromethyl radical C. Afterwards, site-selective addition of radical C to heteroarene 26 yields radical intermediate D, which is subsequently oxidized by Ru^III^ to form the corresponding carbocation E. Finally, deprotonation of intermediate E by a fluoroacetate anion delivers the desired fluoromethylated product 27.

**Scheme 16:**
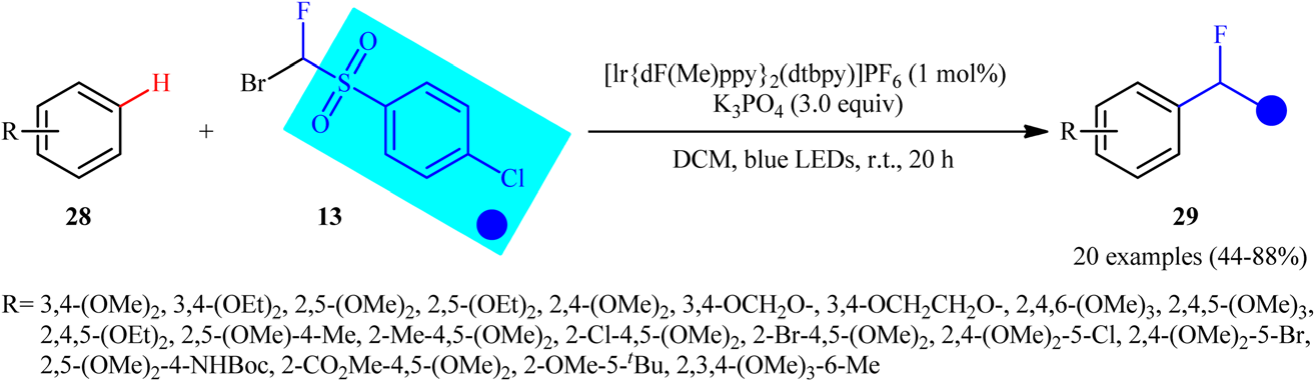
Zhao᾿s synthesis of monofluoromethylated arenes 29.

Along this line, very recently, Zhao and co-workers described that a diverse range of electron-rich arenes 28 were converted to the corresponding monofluoromethylated arenes 29, *via* direct C–H monofluoromethylation with 1-((bromofluoromethyl)sulfonyl)-4-chlorobenzene 13 using [lr{dF(Me)ppy}_2_(dtbpy)]PF_6_ as the photocatalyst under blue LED irradiation at room temperature (Scheme 16).^36^ Notably, a broad array of heteroaromatic substrates, including furan, thiophene, pyrrole, pyridine, pyrazine, and pyrimidine derivatives, were also successfully fluoromethylated under identical conditions. In addition, this protocol enabled the late-stage direct monofluoromethylation of several bioactive molecules, such as bezafibrate, gemfibrozil, propham, and chlorpropham. Importantly, subsequent treatment with Mg efficiently removed the sulfonyl group to deliver the desired monofluoromethylated products (–CH_2_F), and this desulfonylation step was compatible with a wide range of substrates, affording moderate to good yields. According to the authors, this monofluoromethylation reaction proceeds *via* a free-radical pathway, with the sulfonyl group acting as an auxiliary group that enhances the reactivity of the monofluoromethyl radicals.

## 5. Conclusion

Owing to its high atom, step, and pot economy, as well as minimal waste generation, direct C–H bond functionalization has emerged as a highly active area of chemical research. As highlighted in this review, the direct monofluoromethylation of C–H bonds has recently attracted considerable attention as an efficient strategy for incorporating the CH_2_F group into organic molecules, enabling the synthesis of biologically important CH_2_F-substituted compounds within single click (Table 1). Despite the significant progress achieved in this field over the past few years, several challenges remain, which are summarized below: (i) the majority of reported examples rely on non-commercial monofluoromethylation reagents; while the development of new reagents is valuable, expanding the utility of existing and particularly commercially available reagents is equally important; (ii) the scope of monofluoromethylation of aliphatic C–H bonds is mainly limited to carbonyl-containing compounds and, in most cases, involves competing *C*- and *O*-functionalization. Therefore, the development of methods enabling monofluoromethylation of unactivated C_(alkyl)_–H bonds, as well as more regioselective protocols favoring *C*-monofluoromethylation over *O*-monofluoromethylation, is highly desirable; (iii) there is a lack of enantioselective C–H monofluoromethylation reactions, which represents an important direction for future research; (iv) no methodologies have yet been developed for the direct C–H monofluoromethylation of acetylenic C–H bonds; thus, protocols enabling the use of terminal alkynes in this chemistry would be highly desirable; and (v) no examples of regio-switchable monofluoromethylation of (hetero)arenes have been reported, which limits the ability to selectively functionalize different positions on heteroarene scaffolds.

**Table 1:** Comparison of results of direct monofluoromethylation of C–H bonds

Ref.	Yield (%)	Number of examples	Conditions	Commercial availability of CH_2_F reagent	CH_2_F reagent	Entry
22	32–75	3	Cs_2_CO_3_ (6 equiv.), MeCN, r.t., 4 h	—	(Fluoromethyl)(phenyl)(2,3,4,5-tetramethylphenyl)sulfonium tetrafluoroborate	1
24	68–95	13	NaH (2 equiv.), THF, r.t., 1 h	—	(Fluoromethyl)(phenyl)(2,4,6-trimethoxyphenyl)sulfonium hexafluorophosphate	2
25	10–91	27	^*t*^BuOLi (2 equiv.), diglyme, r.t., 2 h	+	ICH_2_F	3
26	26–88	24	[Ru(phen)_3_]Cl_2_ (2 mol%), DNB (1.5 equiv.), Et_3_N (1.5 equiv.), DCM, blue LEDs, r.t., 48–72 h	—	β-Fluorinated *gem*-diols	4
27	38–83	24	Co-(dmgH)_2_(DMAP)Cl (10 mol%), DIPEA (5 equiv.), DCM, blue LEDs, r.t., 24 h	—	1-((Bromofluoromethyl)sulfonyl)-4-chlorobenzene	5
28	56–80	5	^*t*^BuOOH (2–3 equiv.), perfluorotoluene, anisole, or DCE: Water (2.5 : 1), 50 °C, 18–48 h	—	Zn(SO_2_CH_2_F)_2_	6
29	20–79	17	^*t*^BuOLi (3 equiv.), DMSO, 40 °C, 2 h	—	[Ph_3_PCH_2_F]^+^I^−^	7
30	8–80	33	CuSO_4_ (10 mol%), bpy (10 mol%), HPO(OMe)_2_ (10 mol%), Na_2_CO_3_ (2 equiv.), DCE, 110 °C, 24 h	—	α-Bromo-α-fluoro carbonyl compounds	8
32	36–88	32	Pd(OAc)_2_ (10 mol%), P(2-furyl)_3_ (22 mol%), Cs_2_CO_3_ (3 equiv.), NBE (3 equiv.), DCE, 80 °C, 12 h	+	ICH_2_F	9
35	22–82	37	Ru(bpy)_3_Cl_2_·6H_2_O (2 mol%), DCE, blue LEDs, r.t., 16 h	—	PhI(OCOCH_2_F)_2_	10
36	44–88	20	[lr{dF(Me)ppy}_2_(dtbpy)]PF_6_ (1 mol%), K_3_PO_4_ (3.0 equiv), DCM, blue LEDs, r.t., 20 h	—	1-((Bromofluoromethyl)sulfonyl)-4-chlorobenzene	11

## Conflicts of interest

There are no conflicts to declare.

## Data Availability

This article is a review and does not report any original experimental data. Therefore, no new data were generated or analyzed, and data sharing is not applicable.
